# Effectiveness of a structured rubric-based assessment in preclinical crown preparation training: a randomized controlled trial

**DOI:** 10.1186/s12903-026-08667-y

**Published:** 2026-05-22

**Authors:** Marie Fung Ying Chen, Mahesh Mundathaje, Srikant Natarajan, Prashant Bajantri, Sandipan Mukherjee

**Affiliations:** 1 162/87, Ghosh Para Road, Barrackpore, North -24PGS, Kolkata, 700120 India; 2 Department of Prosthodontics and Crown & Bridge, Manipal College of Dental Science Mangalore, Manipal Academy of Higher Education, Manipal, India; 3 Department of Oral Pathology, Manipal College of Dental Science Mangalore, Manipal Academy of Higher Education, Manipal, India

**Keywords:** Dental education, Preclinical training, Crown preparation, Analytical rubric, Competency-based education, Objective assessment, Psychomotor skills, Student feedback, Sustainable Development Goals (SDG 3: Good Health and Well-being; SDG 4: Quality Education)

## Abstract

**Background:**

Crown preparation is a core but challenging skill for dental students, often assessed through subjective methods with limited reliability. Analytical rubrics provide a structured and transparent approach to teaching and evaluation. This study assessed the effectiveness of a rubric-based assessment system in preclinical crown preparation training.

**Methods:**

A randomized controlled trial was conducted with 90 fourth-year undergraduate dental students who met predefined eligibility criteria (mean age: 22 ± 1.2 years). Participants were randomly allocated in a 1:1 ratio using a computer-generated sequence to a control group (*n = *45) evaluated conventionally and a test group (*n = *45) evaluated using a structured rubric. Both groups prepared an all-ceramic crown on tooth #21 following standard instruction. The primary outcome was the total crown preparation score. Assessments were performed independently by calibrated examiners at baseline and post-training; student blinding was not feasible. No adverse events were reported. Independent and paired t-tests with Bonferroni correction were applied, effect sizes calculated, and post hoc power analysis performed using a 95% confidence interval. Student perceptions were collected using a six-item Likert-scale questionnaire.

**Results:**

Baseline comparisons had no significant differences (*p = *1.0). At the end of training, the primary outcome show, test group demonstrated significantly higher scores across all parameters (all *p* < 0.001). Mean total score was 30.53 ± 4.99 in the test group versus 10.56 ± 4.34 in the control group (*p* < 0.001; Cohen’s d = 4.21). Both groups improved significantly, with greater gains in the test group (22.91 ± 6.42 vs 2.93 ± 2.72). Post hoc power exceeded 0.99 for all outcomes. Student feedback was highly positive, with 95% affirming improved objectivity, self-reflection, and clarity of evaluation criteria.

**Conclusions:**

The use of an analytical rubric was associated with higher crown preparation assessment scores and positive student perceptions regarding clarity and objectivity of evaluation. These findings suggest that rubric-based assessment may support structured performance evaluation and learner understanding in preclinical prosthodontic training.

**Trial registration:**

Not registered. This study evaluated an educational assessment intervention in a preclinical academic setting and did not involve patients or health-related clinical interventions as defined under ICMJE criteria.

## Introduction

Learning to prepare a crown is a foundational and challenging skill for dental students, as it requires a high degree of psychomotor, cognitive, and communication abilities [1]. Traditionally, the assessment of these skills in preclinical courses has relied on subjective, empirical, or global grading methods, which can suffer from inconsistencies and a lack of objectivity among instructors [2, 3]. This subjectivity can lead to student frustration, as the criteria for a good performance may not be clear [4].

To address these challenges, there has been a growing emphasis on more objective and transparent assessment tools, such as rubrics [4, 5]. A rubric is a structured scoring guide with defined criteria and performance levels, which can enhance the objectivity, transparency, and consistency of evaluations [2, 5, 6]. Research has shown that using rubrics can improve both inter- and intra-rater reliability, ensuring that different examiners and the same examiner over time can consistently evaluate student work [6]. This is particularly important in preclinical courses where different faculty members may be involved in the assessment process [2].

Furthermore, rubrics are not merely a grading tool; they are a pedagogical instrument that can support student learning [7]. They make learning goals and expectations explicit for both students and instructors, helping students to better understand what is required for a high-level performance [4, 8]. This transparency can empower students to engage in self-reflection and self-assessment, allowing them to identify their own strengths and weaknesses and take a more active role in their learning [7, 9, 10]. The use of rubrics has also been found to improve learning motivation and career interest among dental students [11].

From an educational perspective, the use of structured analytical rubrics aligns with principles of self-regulated learning and constructive alignment. Self-regulated learning emphasizes learners’ active engagement in goal setting, self-monitoring, and reflection, processes that are facilitated when assessment criteria are explicit and transparent. Analytical rubrics make performance expectations visible, enabling students to evaluate their own work against defined standards and adjust their learning strategies accordingly. Furthermore, by aligning instructional objectives, learning activities, and assessment criteria, rubric-based assessment supports constructive alignment, which has been shown to enhance skill acquisition in preclinical dental education. Grounding the present intervention within these pedagogical frameworks provides a theoretical basis for examining how structured assessment can influence preclinical crown preparation performance [12].

Although analytical rubrics have been widely adopted in health professions education, much of the existing evidence is derived from descriptive studies, self-reported perceptions, or non-randomized designs. There remains limited high-quality experimental evidence demonstrating the causal impact of rubric-based assessment on objectively measured psychomotor skill acquisition in preclinical dental training. In particular, few studies have employed randomized controlled designs with baseline performance assessment to evaluate the effect of rubrics on complex operative procedures such as crown preparation. The present study addresses this gap by examining the effectiveness of a structured rubric-based assessment system on preclinical crown preparation performance using a randomized controlled methodology with objective outcome measures.

## Materials and methods

### Study design and setting

This study employed a randomized controlled trial design to investigate the effectiveness of an analytical assessment system in preclinical crown preparation training. A total of 90 fourth-year undergraduate dental students were randomly and equally divided into two groups: a control group (*n = *45) and a test group (*n = *45) [13]. All participants were engaged in the preparation of an all-ceramic crown on tooth #21 as part of their fixed prosthodontics curriculum [14].

### Sample size and power considerations

The sample size was determined by the number of eligible fourth-year undergraduate dental students available during the study period and was therefore fixed by the academic cohort rather than by an a priori power calculation. Following completion of data collection, a post hoc power analysis was performed using G*Power software (version X.X; Universität Düsseldorf, Germany) to evaluate whether the study was adequately powered to detect differences between groups. Based on the observed means and standard deviations for the primary outcome, the achieved statistical power exceeded 0.99 at an alpha level of 0.05. Power remained > 0.99 even after adjustment for multiple comparisons using Bonferroni correction.

### Trial registration

This randomized controlled educational study was conducted in a preclinical simulation laboratory setting among undergraduate dental students. The intervention pertained to assessment methodology and did not involve patient care, therapeutic procedures, or health-related clinical outcomes.

### Participants and eligibility criteria

The study cohort comprised Indian fourth-year undergraduate dental students enrolled in the preclinical prosthodontics course during the study period. The mean age of participants was 22.0 ± 1.2 years, with 69 females and 21 males included. All participants were from the same academic year and followed an identical preclinical curriculum.

Inclusion criteria were: (1) enrolment in the fourth year of the undergraduate dental program, (2) completion of prerequisite preclinical training in tooth preparation, and (3) willingness to participate in the study.

Exclusion criteria included: (1) prior clinical experience in fixed prosthodontics beyond the prescribed undergraduate curriculum, (2) absence during baseline assessment or training sessions, and (3) incomplete crown preparation or missing assessment data.

### Randomization and allocation concealment

Eligible participants were randomly allocated to either the control group or the rubric-based assessment group in a 1:1 ratio using a computer-generated randomization sequence. Allocation was implemented by an independent administrative staff member using the computer-generated sequence, and group assignment was disclosed only after baseline assessment. Group assignment was revealed to participants only after completion of baseline assessment. Due to the educational nature of the intervention, blinding of participants was not feasible. However, outcome assessments were performed independently by calibrated examiners who were not involved in the instructional process.

### Baseline and post-training crown preparation assessments

Following randomization and prior to the training intervention, all participants completed a baseline crown preparation for an all-ceramic crown on typodont tooth #21 under standardized simulation conditions. This preparation represented the pre-training performance measure.

After completion of the instructional and practice sessions, all students performed a second crown preparation on the same tooth (#21), which served as the post-training assessment.

Both baseline and post-training preparations were independently evaluated by two calibrated faculty examiners using identical assessment criteria to ensure comparability across time points.

### Assessment tool and procedure

Before the study began, all students received standard theoretical and practical instruction on crown preparation techniques. Students in the test group were also provided with a clear, structured rubric system that outlined the specific criteria for assessing their work [15]. This rubric was adapted from previous studies that have demonstrated its utility in enhancing the reliability of dental student evaluations [6].

### Description and development of the rubric

The analytical rubric was adapted from an existing rubric framework originally designed to support structured assessment and feedback in educational settings. For the present study, the rubric was substantially modified to align with the psychomotor and technical requirements of preclinical crown preparation. Narrative descriptors were replaced with procedure-specific criteria, including incisal reduction, axial reduction, finish line configuration, line angle rounding, and ergonomics. Performance descriptors were rewritten to reflect observable operative outcomes rather than written or reflective components, ensuring relevance to technical skill assessment in prosthodontics.

Content relevance and clarity of the adapted rubric were reviewed by a panel of six subject-matter experts with experience in prosthodontics and preclinical dental education. The experts evaluated each criterion for appropriateness, clarity, and alignment with preclinical learning objectives. Feedback from the panel was used to refine wording, remove ambiguity, and ensure that each criterion reflected expected competency levels for undergraduate dental students.

Although a numerical Content Validity Index (CVI) was not calculated, the structured expert review process provided qualitative evidence of content validity, supporting the appropriateness of the rubric for assessing technical crown preparation skills.

### Examiner calibration and assessment procedure

Two faculty examiners with experience in preclinical prosthodontic teaching independently evaluated all crown preparations. Prior to data collection, the examiners underwent a calibration session in which sample preparations were jointly reviewed and discussed to achieve consensus on rubric interpretation and scoring criteria as shown in Table 1. During the study, examiners were blinded to group allocation and assessment time point. In cases of discrepancy, scores were discussed and resolved by consensus to ensure consistency in evaluation. The same evaluators scored all preparations at two distinct time points: once at the beginning of the training session and again at the end to evaluate skill acquisition and improvement. The assessments were recorded digitally and used as the primary data for analysis [16].

**Table 1:** Structured rubrics used for assessment of crown preparation

Parameter	Maximum Score	Properly Done (Full Score)	Partially Done (Half Score)	Not Done (No Score)
Incisal tooth reduction	6	Uniform reduction of 2 mm with incisal bevel	Moderate reduction < 1.5 mm	Reduction < 1.5 mm or irregular
Proximal tooth reduction (mesial & distal axial walls)	8	Angulation within 10° on each side	Angulation up to 15°	Angulation > 20°
Labial tooth reduction	8	Two-plane reduction without undercuts	Two-plane reduction with minimal undercuts	No two-plane reduction/with undercuts
Lingual tooth reduction	8	Proper path of insertion with maintained cingulum	Path of insertion not maintained; cingulum not reduced	Over-reduction of cingulum
Finish line width & finishing	4	Equal finish line width, smooth surface	Uneven/irregular finish line	Width > 2 mm
Point angles & line angles	4	Rounded angles, smooth transition of axial walls	Uneven walls with sharp angles	Rough, over-contoured surface
Ergonomics (operator & phantom head position)	2	Optimum position maintained	Acceptable with minor bending	Incorrect position
**Total Score**	**40**			

### Statistical analysis

A post hoc power analysis was performed to ensure the sample size was sufficient to detect a significant effect. Statistical analysis was performed using SPSS software (Version 28.0; IBM, Chicago, IL). Descriptive statistics, including means and standard deviations, were calculated for all variables. Normality of the data was assessed using the Shapiro–Wilk test, and homogeneity of variance was confirmed with Levene’s test.

Independent t-tests were used to compare the mean scores between the control and test groups [3]. Within-group changes from baseline to post-training were analysed using paired t-tests, as each participant contributed two related observations (pre-training and post-training crown preparations). A Bonferroni correction was applied to all analyses to adjust for multiple comparisons, with a significance level set at p < 0.05. Effect sizes were reported using Cohen’s d [2].

### Student feedback and ethical considerations

Student perceptions of the rubric-based assessment were collected using a six-item Likert-scale questionnaire adapted from a previously validated instrument reported by Ali et al. [17]. The original questionnaire was designed to evaluate educational interventions and learner perceptions in undergraduate dental education. For the present study, items were contextually modified to reflect rubric-based assessment in preclinical crown preparation, while retaining the underlying constructs related to clarity of assessment, self-reflection, and learning support. As the instrument was derived from an established and validated tool, content relevance was considered appropriate for exploratory evaluation of student perceptions in this context.

The study was conducted in accordance with the ethical guidelines of the Declaration of Helsinki and received approval from the Institutional Ethics Committee (Ref. No. 125/2024). All participants provided written informed consent.

### Protocol deviations and stopping guidelines

No changes were made to the study design, intervention, outcomes, or assessment procedures after trial commencement. Given the educational nature of the intervention and the absence of anticipated risks, no stopping guidelines were defined.

## Results

**Fig. 1:**
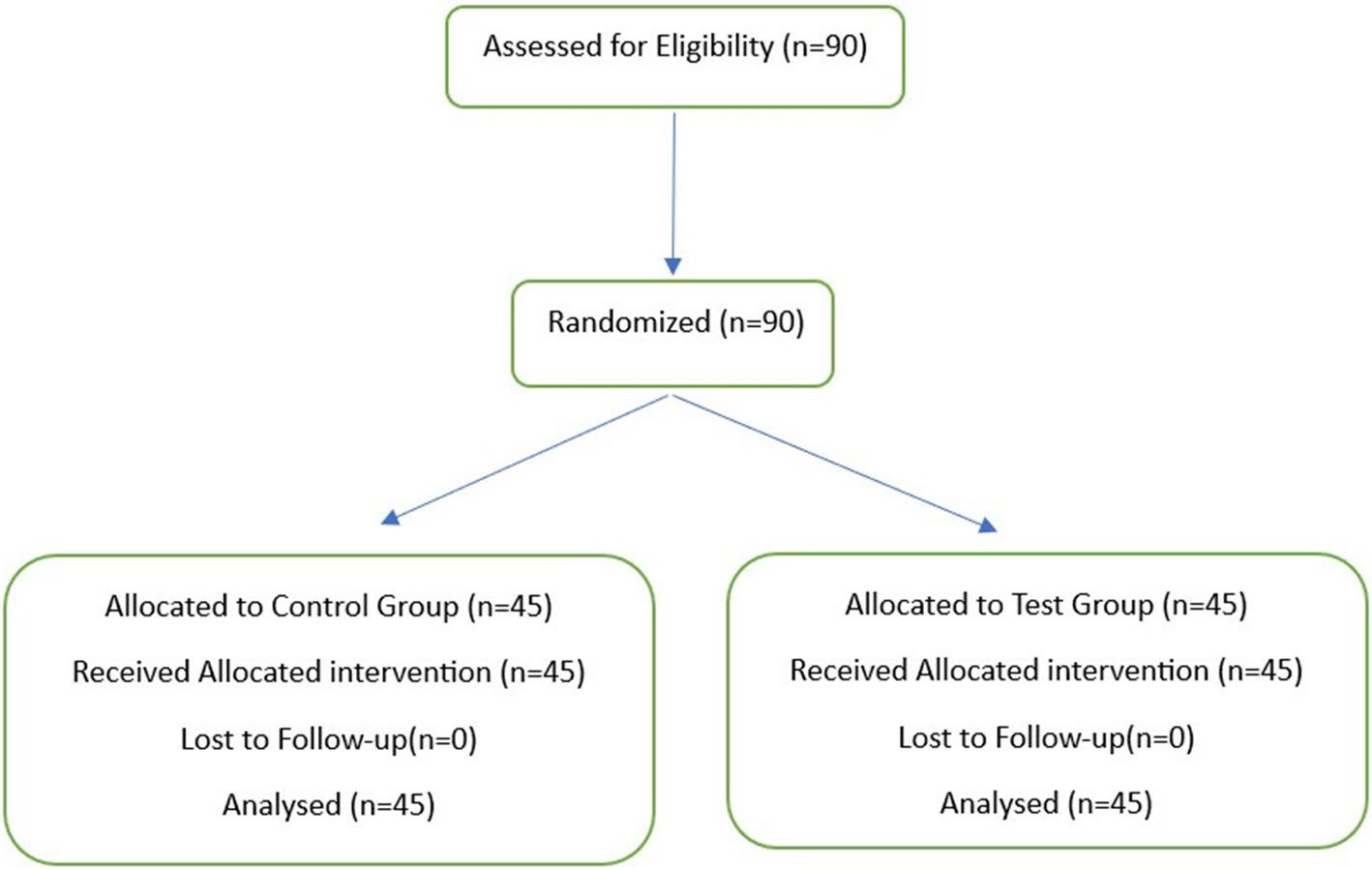
CONSORT flow diagram of participant recruitment, randomization, allocation, and analysis

**Fig. 2:**
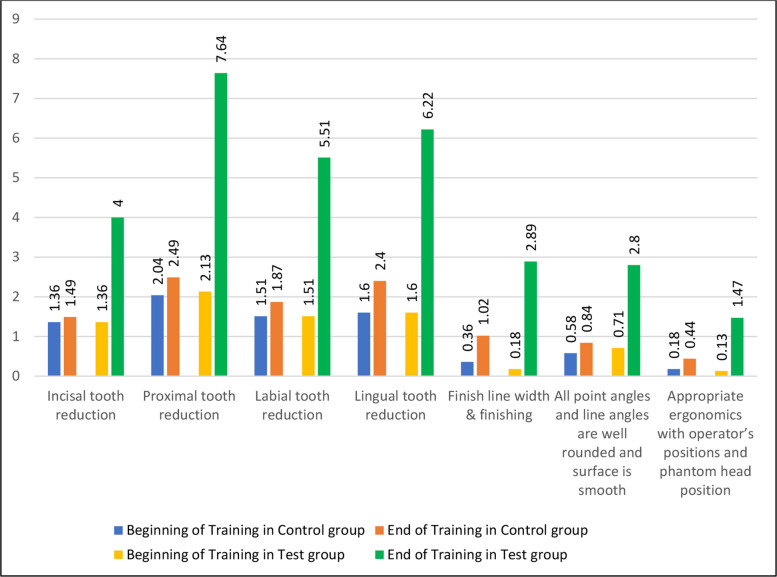
Comparison of mean scores for all crown preparation parameters between control and test groups at the end of training

**Fig. 3:**
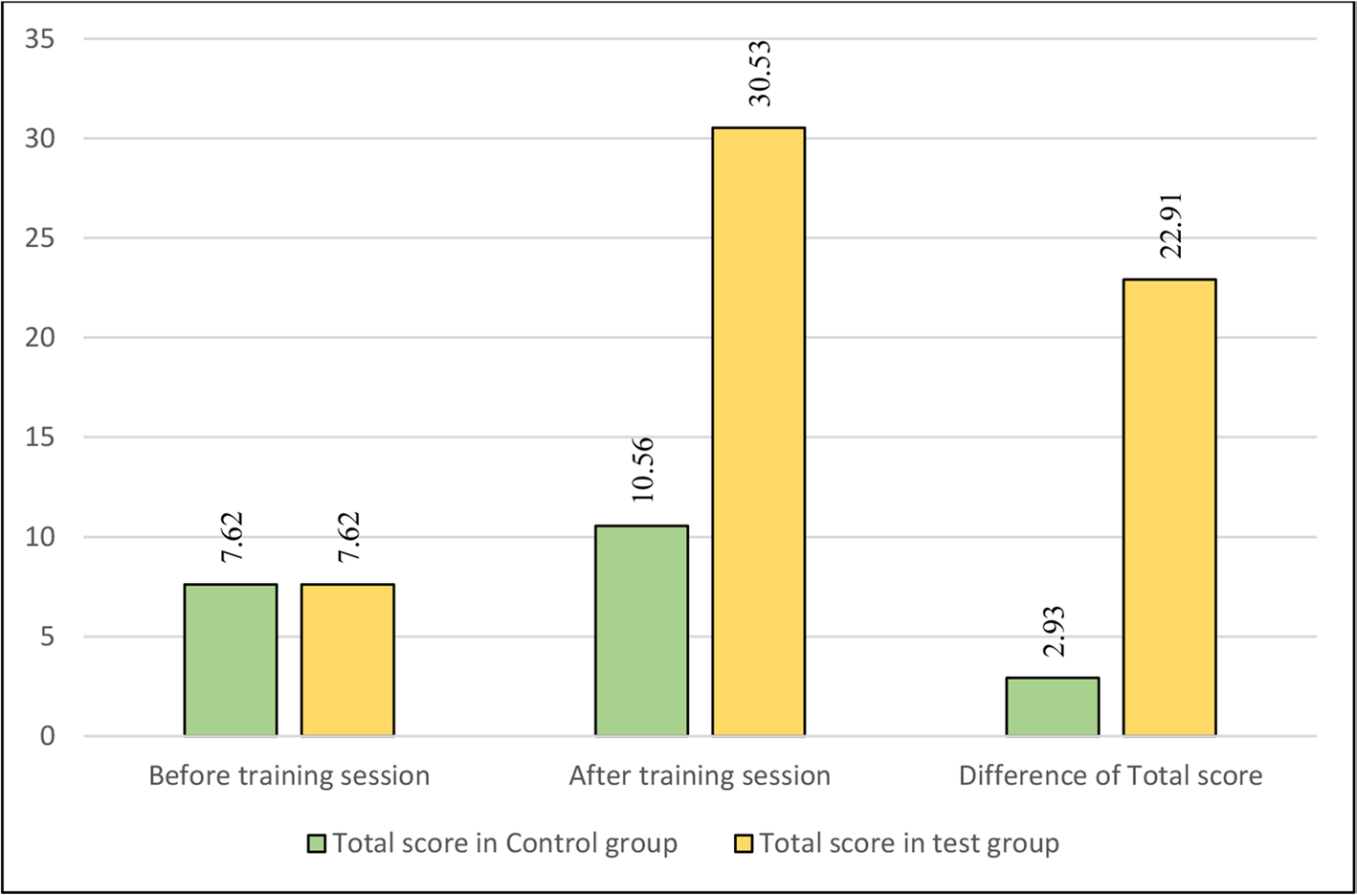
Comparison of total crown preparation scores between control and test groups at the end of training

Participant recruitment, randomization, allocation, and analysis are summarized in the CONSORT flow diagram Fig. 1. A total of 90 fourth-year undergraduate dental students completed the study. Analysis of the data showed that both the control and test groups were comparable at the beginning of the training session, with no statistically significant differences in their baseline mean scores for any of the assessed parameters. At baseline, no statistically significant differences were observed between the control and test groups for any assessed parameter (all p > 0.05). Mean baseline scores were similar across groups, reflecting comparable initial skill levels following randomization. Although baseline means appeared numerically identical for certain parameters, variability was present, as reflected by the standard deviations reported in Table 2. This clustering of scores is consistent with novice performance in early preclinical crown preparation exercises. For instance, parameters such as incisal tooth reduction, labial tooth reduction, and lingual tooth reduction had nearly identical mean scores with a statistically non-significant p-value of 1.0 as detailed in Table 3 and Fig. 2. While the control group had slightly higher initial scores for finish line width, finishing, and ergonomics, these differences were not statistically significant (*p = *0.22 and *p = *0.596, respectively). The total scores at the beginning of the training session also reflected this parity, with both groups having a mean score of 7.62 (*p = *1.0), as shown in Fig. 3.

**Table 2:** Within-group comparison of baseline and post-training crown preparation scores using paired t-tests

Parameters	Pre- training in Control	Pre- training in Test	Post training in Control	Post training in Test
Incisal tooth reduction	1.36	1.36	1.49	4
Proximal tooth reduction	2.04	2.13	2.49	7.64
Labial tooth reduction	1.51	1.51	1.87	5.51
Lingual tooth reduction	1.6	1.6	2.4	6.22
Finish line width & finishing	0.36	0.18	1.02	2.89
All point angles and line angles are well rounded, and surface is smooth	0.58	0.71	0.84	2.8
Appropriate ergonomics with operator's positions and phantom head position	0.18	0.13	0.44	1.47

**Table 3:** Post-training comparison between groups (independent t-test)

Outcome	Control (Mean ± SD)	Test (Mean ± SD)	*T*	*p*-value	Cohen’s *d*
Incisal tooth reduction	1.49 ± 1.79	4.00 ± 1.92	–6.417	< 0.001	1.34
Proximal tooth reduction (mesial & distal axial walls)	2.49 ± 2.14	7.64 ± 1.15	–14.239	< 0.001	2.91
Labial tooth reduction	1.87 ± 2.02	5.51 ± 2.46	–7.692	< 0.001	1.58
Lingual tooth reduction	2.40 ± 1.98	6.22 ± 2.01	–9.084	< 0.001	1.91
Finish line width & finishing	1.02 ± 1.10	2.89 ± 1.01	–8.415	< 0.001	1.77
Point & line angles rounded & smooth	0.84 ± 1.00	2.80 ± 0.99	–9.323	< 0.001	2.00
Ergonomics	0.44 ± 0.50	1.47 ± 0.55	–9.225	< 0.001	1.94
Total Score	10.56 ± 4.34	30.53 ± 4.99	–20.286	< 0.001	4.21

### Primary outcome: Total crown preparation score

The total score difference between the pre- and post-training sessions was also highly significant for both groups. However, the magnitude of the improvement was substantially greater in the test group, with a mean difference of 22.91 ± 6.42 compared to the control group's mean difference of 2.93 ± 2.72. The total post-training mean score for the test group was 30.53 ± 4.99, which was significantly higher than the control group's score of 10.56 ± 4.34 (*p* < 0.001) as depicted in Fig. 3.

### Secondary outcomes: Individual crown preparation parameters

At the conclusion of the training session, a marked difference was observed. The test group, which utilized the structured rubric, demonstrated significantly higher mean scores across all parameters compared to the control group (Table 3, Fig. 2). The observed effect sizes ranged from large to very large, reflecting substantial differences in post-training performance between groups. For instance, incisal tooth reduction in the test group had a mean score of 4.0 ± 1.92 compared to 1.49 ± 1.79 in the control group, a difference that was highly statistically significant (*p* < 0.001). Similar highly significant improvements were seen in the test group for proximal tooth reduction (*p* < 0.001), labial tooth reduction (*p* < 0.001), and lingual tooth reduction (*p* < 0.001). The other three parameters—finish line width and finishing, all point angles and line angles well rounded, and appropriate ergonomics—also showed statistically significant improvements in the test group compared to the control group, all with *p*-values of < 0.001 as documented in Table 3 and Fig. 2.

A paired t-test was conducted to evaluate the change in scores from the beginning to the end of the training session within each group as shown in Tables 2. In the control group, while most individual factors showed improvement, only three of the four were statistically significant. In contrast, the test group showed statistically significant improvement across all individual factors, indicating a more comprehensive and effective learning process.

### Secondary outcomes: Student feedback

Student feedback on the analytical assessment system was overwhelmingly positive. As shown in the overall percentages in Table 4, the highest percentage of responses fell into the 'Strongly Agree' category (59.63%), followed by 'Agree' (35.18%). The feedback also revealed that students found the rubric most helpful for self-reflection (66.67% strongly agreed) and for objectively understanding how they would be evaluated (62.22% strongly agreed). The ability to identify each step and ensure impartial assessment also received high marks, with 57.78% of students in each category selecting 'Strongly Agree.' The detailed breakdown of student responses for each question is provided in Table 5. Student feedback indicated a high level of agreement regarding the perceived usefulness and clarity of the rubric-based assessment approach.

**Table 4:** Student feedback summery

Criteria	No. of response to QA	No. of response to QB	No. of response to QC	No. of response to QD	No. of response to QE	No. of response to QF	Sum total	Overall % of each criterion
Strongly agree	26	28	30	27	24	26	161	59.63%
Agree	18	17	11	12	20	17	95	35.18%
Neutral	1	0	4	6	1	2	14	5.18%
Strongly Disagree	0	0	0	0	0	0	0	0%
Disagree	0	0	0	0	0	0	0	0%

**Table 5:** Detailed student feedback

Criteria	QA	QB	QC	QD	QE	QF
% for Strongly agree	57.78	62.22	66.67	60	53.33	57.78
% for Agree	40	37.78	24.44	26.67	44.44	37.78
% for Neutral	2.22	0	8.89	13.33	2.22	4.44
% for Disagree	0	0	0	0	0	0
% for Strongly Disagree	0	0	0	0	0	0

### Post hocpower analysis

A post hoc power analysis was conducted for all assessed parameters using the observed means and standard deviations. All outcomes demonstrated large to very large effect sizes (Cohen’s *d* = 1.34–4.21). The statistical power achieved was greater than 0.99 for all comparisons at α = 0.05. Even after applying Bonferroni correction for multiple testing (α = 0.00625 for eight outcomes), power remained > 0.99. Detailed post hoc power values for each outcome are presented in Table 6.

**Table 6:** Post hoc power analysis for assessed outcomes

Outcome	Cohen's d	Post hoc Power (α = 0.05)	Post hoc Power (Bonferroni α = 0.00625)
Incisal tooth reduction	1.34	> 0.99	> 0.99
Proximal tooth reduction (mesial & distal axial walls)	2.91	> 0.99	> 0.99
Labial tooth reduction	1.58	> 0.99	> 0.99
Lingual tooth reduction	1.91	> 0.99	> 0.99
Finish line width & finishing	1.77	> 0.99	> 0.99
Point & line angles rounded & smooth	2.0	> 0.99	> 0.99
Ergonomics	1.94	> 0.99	> 0.99
Total Score	4.21	> 0.99	> 0.99

## Discussion

The implementation of an analytical rubric for pre-clinical crown preparation in this study yielded significant improvements in student performance, aligning with established principles of effective dental education. Historically, objective grading systems have been advocated to enhance the evaluation process, with Dhuru et al. pioneering a criteria-oriented grading system for preclinical courses as early as 1978 [3]. This foundational idea has been consistently supported by contemporary research, which emphasizes that well-designed analytical rubrics can improve the objectivity and consistency of evaluations, while also providing students with detailed, constructive feedback [2, 6, 16]. The primary outcome of this study was improvement in rubric-based assessment scores and student perceptions, rather than direct measurement of long-term learning or skill retention. Our findings directly support this pedagogical approach. The independent t-test results demonstrated that while the control and test groups were comparable at the start of the training, the test group's scores showed a statistically significant improvement across all assessed parameters at the end of the session. This outcome underscores the tangible benefits of a structured, criteria-based learning environment. Such results are consistent with other studies that have shown the positive impact of rubrics on dental students' learning and technical skill acquisition, suggesting that clear evaluation criteria empower students to understand and master complex psychomotor tasks [2, 15, 18].

The detailed analysis of individual parameters revealed specific areas of learning enhancement. Among the individual parameters, proximal tooth reduction showed the greatest relative improvement in the rubric group, suggesting that structured criteria may be particularly effective for guiding students through technically demanding steps of crown preparation. This finding suggests that the rubric was particularly effective in guiding students through this technically challenging aspect of crown preparation. This contrasts with a previous study by Habib et al. (2018), where proximal reduction was identified as a common area of weakness, receiving the lowest mean scores [13]. The improved performance in our study highlights the rubric's effectiveness in targeting and remediating specific skill deficiencies. The least improvement was observed in appropriate ergonomics, which may reflect the relative complexity of modifying ergonomic behaviours within a short training period.

The very large effect sizes observed in this study, particularly for total crown preparation scores, should be interpreted within the specific context of preclinical skill acquisition. Participants demonstrated low and clustered baseline performance typical of novice learners, while the structured rubric provided explicit performance criteria that substantially increased post-training scores in the intervention group. This combination of restricted baseline variability, a bounded scoring scale, and a targeted educational intervention applied to a discrete psychomotor task may contribute to inflated standardized effect size estimates. Similar patterns of large effect sizes have been reported in controlled educational interventions assessing early-stage technical skill development.

The qualitative feedback from the students corroborated the quantitative findings. Most students strongly agreed that the rubric helped them to reflect on their work objectively, which is a crucial skill for a self-regulated lifelong learner [19]. This is particularly important because self-assessment and reflection are foundational competencies required for clinical practice. The high percentage of students who felt the rubric helped them understand how they would be evaluated objectively and improve their scores effectively further solidifies the role of analytical rubrics in fostering a transparent and supportive learning environment [20]. The fact that a small percentage of students remained neutral regarding the rubric's help in rectifying their work.

suggests that, while highly effective, the rubric alone may not suffice for all students, and supplementary feedback from instructors remains essential. The student feedback questionnaire was adapted from a previously validated instrument; however, formal psychometric validation, including internal consistency and reliability testing, was not performed in the present cohort. Consequently, student feedback findings should be interpreted as exploratory and descriptive rather than confirmatory. Future studies should undertake full validation of perception instruments specific to rubric-based assessment in preclinical dental education.

### Contribution to evidence

While the use of rubrics in dental education is well established, the present study extends existing evidence in several important ways. Unlike many prior studies that relied primarily on student perceptions or observational designs, this randomized controlled trial incorporated baseline performance assessment, examiner blinding, and objective evaluation of psychomotor outcomes. The findings demonstrate not only learner acceptance of rubric-based assessment but also a substantial and quantifiable improvement in technical performance. By applying rubric-based assessment to crown preparation—a procedure requiring precise spatial judgment and fine motor control—this study provides experimental evidence supporting the role of structured rubrics as active learning scaffolds in preclinical prosthodontic training rather than merely as grading instruments.

The findings of this study must be interpreted within its limitations. The study was conducted at a single dental institution and, as such, the results may not be generalizable to other settings. The sample size, while adequate for statistical analysis, could be expanded in future studies. The absence of prospective trial registration is acknowledged as a limitation and may restrict external verification of protocol adherence. While the rubric underwent expert review to establish content validity, psychometric properties such as internal consistency and inter-rater reliability were not formally quantified. Future studies should incorporate statistical validation measures, including reliability testing and factor analysis, to further strengthen the measurement properties of rubric-based assessment tools in preclinical dental education. Another limitation of this study is that formal inter- and intra-examiner reliability statistics, such as intraclass correlation coefficients, were not calculated. Although examiners were calibrated prior to assessment and followed standardized scoring criteria, the absence of quantified reliability measures may limit the generalizability of the findings. Future studies should incorporate formal reliability testing to further strengthen the methodological rigor of rubric-based assessment in preclinical dental education. A further limitation relates to the magnitude of the observed effect sizes. While Cohen’s d values were mathematically correct, standardized effect sizes can appear inflated in educational studies involving novice learners, limited score ranges, and rubric-based assessments. Therefore, the effect sizes reported should be interpreted as indicators of strong educational impact within the study context rather than as estimates generalizable across curricula or institutions.

Although improved post-training scores were observed in the rubric group, the present findings should be interpreted in the context of assessment performance rather than definitive learning gain. The study demonstrates the potential value of rubric-based assessment in enhancing transparency, standardization, and student understanding of evaluation criteria within preclinical crown preparation exercises. Future longitudinal studies incorporating repeated training cycles and validated learning outcome measures are required to establish sustained educational impact.

## Conclusion

Based on the study's findings, the analytical assessment system proved to be an effective educational tool in preclinical crown preparation training for undergraduate dental students. The introduction of structured rubrics led to a significant improvement in the technical skills of students in the test group, as evidenced by their statistically higher mean scores after the training session.

Ultimately, the system not only improved student performance but also fostered essential skills such as self-reflection and objective self-assessment. The positive feedback from the students confirmed that the rubric-based approach enhanced their understanding of the evaluation criteria, allowing them to identify their strengths and weaknesses more effectively. This suggests that incorporating such analytical systems into dental education curricula can lead to more competent, confident, and self-directed learners. Beyond confirming the general utility of rubrics, this study provides randomized evidence that structured analytical rubrics can meaningfully enhance the acquisition of complex psychomotor skills in preclinical prosthodontics, thereby contributing to evidence-based educational practice in dental training.

## Data Availability

The datasets generated and/or analyzed during the current study are available from the corresponding author on reasonable request.
